# Psychological Distress in Family Members of Individuals with Autism Spectrum Disorder—Associated Factors and Implications for Healthcare

**DOI:** 10.3390/bs16071175

**Published:** 2026-07-11

**Authors:** Piotr Bromber, Mariola Borowska, Beata Gellert, Natalia Miller, Maria Malm, Agnieszka Drab, Janusz Ostrowski, Jarosław Pinkas, Wojciech Miazga, Anna Augustynowicz, Urszula Religioni

**Affiliations:** 1 School of Public Health, Centre of Postgraduate Medical Education of Warsaw, 01-813 Warsaw, Poland; piotr.bromber@cmkp.edu.pl (P.B.); beata.gellert@cmkp.edu.pl (B.G.); janusz.ostrowski@cmkp.edu.pl (J.O.); jaroslaw.pinkas@cmkp.edu.pl (J.P.); wojciech.miazga@cmkp.edu.pl (W.M.); anna.augustynowicz@cmkp.edu.pl (A.A.); urszula.religioni@cmkp.edu.pl (U.R.); 2 Department of Environmental Threat Prevention, Allergology and Immunology, Medical University of Warsaw, 01-813 Warsaw, Poland; natalia.miller@wum.edu.pl; 3 Department of Medical Informatics and Statistics, Medical University of Lublin, 20-059 Lublin, Poland; maria.malm@umlub.edu.pl (M.M.); agnieszka.drab@umlub.pl (A.D.)

**Keywords:** autism spectrum disorder, ASD, family caregivers, psychological distress, healthcare system, public health

## Abstract

Background: Autism spectrum disorder (ASD) affects not only diagnosed individuals but also their families, who often experience long-term psychological and organizational burden. This study aimed to assess depression, anxiety, and stress among family members of individuals with ASD and to identify socio-demographic and family-related associated factors of psychological distress, with particular attention to high-risk groups, social inequalities, and the healthcare system context. Methods: A cross-sectional study was conducted in February 2025 among 310 family members of individuals with ASD. Data were collected using the Computer-Assisted Web Interview (CAWI) method via the Ariadna Nationwide Research Panel. Psychological distress was assessed using the Depression Anxiety Stress Scales (DASS-21). Results: Respondents reported substantial levels of psychological distress. Severe or extremely severe symptoms were most common for anxiety (41.29%), followed by depression (35.48%) and stress (27.74%). Younger respondent age was consistently associated with higher depression, anxiety, and stress. Lower income was associated with higher depression and anxiety, indicating the importance of socio-economic inequalities. Conclusions: The findings highlight the need to identify high-risk groups and develop family-centered, coordinated support within the healthcare system, with particular attention to social inequalities, continuity of care, and access to psychological and therapeutic services.

## 1. Introduction

Autism spectrum disorder (ASD) represents a complex group of neurodevelopmental conditions with growing epidemiological and societal significance. It is estimated that ASD affects approximately 1–2% of the pediatric population, making it one of the key challenges in contemporary public health. Increasingly, the understanding of ASD extends beyond a purely clinical perspective to encompass its broader impact on the entire family system. Families of individuals with ASD play a central role in care, therapy, and support, which is associated with long-term psychological and organizational burden (Warreman et al., 2023; Boulton et al., 2024).

Available evidence indicates that family members of individuals with ASD, particularly caregivers, experience higher levels of depression, anxiety, and stress compared to the general population (Wang, 2021; Bischof et al., 2018; Schnabel et al., 2020). This burden is typically chronic and results from the accumulation of multiple factors, including caregiving intensity, behavioral challenges associated with ASD, limited access to support services, and uncertainty about the future (Bischof et al., 2018). At the same time, psychological distress in this population is heterogeneous and influenced by individual and socio-economic characteristics (Ault et al., 2021).

Among the most frequently examined determinants are socio-demographic factors such as gender, educational level, and financial situation. Previous studies have shown that women—especially mothers—are more likely to report higher levels of stress and depressive symptoms than men (Li et al., 2022). Economic status also plays a significant role, as lower income and restricted access to resources may increase the risk of poorer mental health outcomes (Wang, 2021; Santiago et al., 2024).

Increasing attention has also been given to relational factors, including the degree of kinship with the individual with ASD. While most research focuses on parents, existing evidence suggests that siblings and other family members may also experience considerable emotional and psychosocial challenges (Jones et al., 2020; Múries-Cantán et al., 2022).

Factors related to the course of ASD within the family are also important. The period surrounding diagnosis and subsequent life stages of the individual with ASD are associated with distinct adaptive challenges. Longitudinal studies indicate that levels of stress and depressive-anxiety symptoms may change over time; however, in some families, they remain persistently high (Rattaz et al., 2023).

Despite the growing body of research, there remains a need for analyses that simultaneously consider multiple dimensions of psychological functioning alongside a broad range of social and system-level determinants. In families of individuals with ASD, psychological distress may be influenced by a range of factors. Although the present study focuses on individual and family characteristics, previous literature suggests that broader contextual factors, including access to services and support systems, may also play a role. An integrated approach that considers depression, anxiety, and stress as related but distinct constructs is particularly important for accurately identifying risk profiles.

The aim of this study was to assess levels of depression, anxiety, and stress among family members of individuals with ASD and to identify socio-demographic and family-related factors associated with higher levels of psychological distress, with particular emphasis on social inequalities, high-risk groups, and implications for the organization of support within the healthcare system.

## 2. Materials and Methods

This cross-sectional study was conducted among family members of individuals diagnosed with autism spectrum disorder (ASD) in February 2025. Data were collected using the Computer-Assisted Web Interview (CAWI) method via the Ariadna Nationwide Research Panel, a large, nationwide online research panel that enables recruitment of a geographically diverse quota-based sample of respondents across Poland. The use of an online data collection method allowed for efficient recruitment of participants and ensured anonymity, which is particularly important when assessing sensitive aspects such as mental health.

Eligible participants were individuals aged 15 years and older who reported having a family member diagnosed with ASD. In this study, a “family member” was defined as an individual who reported having a relative or spouse diagnosed with ASD and who lived in the same household as that person. This included parents, siblings, grandparents, grandchildren, spouses, and other relatives. The inclusion of various types of family relationships (e.g., parents, siblings, extended family members) allowed for a broader assessment of psychological burden within the family system.

The research instrument consisted of a structured questionnaire divided into several sections. The first section included socio-demographic variables such as gender, age, education level, employment status, and average monthly income per household member. The second section included family-related variables, including the degree of kinship with the individual with ASD. The third section collected information about the individual with ASD, specifically age and time elapsed since diagnosis.

Psychological distress was assessed using the Depression Anxiety Stress Scales (DASS-21), a widely used and validated self-report instrument designed to measure three related but distinct dimensions: depression, anxiety, and stress. Each subscale consists of seven items rated on a four-point Likert scale, reflecting the extent to which respondents experienced each symptom over the past week. The DASS-21 allows for both continuous assessment and categorization into severity levels (normal, mild, moderate, severe, and extremely severe), providing a comprehensive evaluation of psychological functioning.

### Statistical Analyses

Statistical analyses were performed using Statistica 14 (Cloud Software Group, Inc., 2023; Data Science Workbench, version 14, http://tibco.com). The characteristics of the study population were described using frequencies and percentages for socio-demographic variables, along with selected descriptive statistics. Results obtained from the DASS-21 scale were presented using measures of central tendency and dispersion, including mean, standard deviation, median, quartiles (first and third), mode, as well as minimum and maximum values. The severity levels of depression, anxiety, and stress were additionally presented as frequencies and percentages.

To examine the relationships between socio-demographic variables and levels of depression, anxiety, and stress, multiple linear regression analysis with backward stepwise selection was applied. The independent variables included respondent gender, respondent age, age of the family member with ASD, degree of kinship with the individual with ASD, education level, employment status, average monthly income per capita, and time since ASD diagnosis.

Categorical variables with more than two categories were transformed into dummy variables. The following reference categories were used: degree of kinship (child), education level (primary education), employment status (employed), average monthly income per capita (<1500), and time since diagnosis (<1 year). In total, the initial model included 20 parameters corresponding to 8 independent variables.

The analysis began with a full model including all predictors. A backward stepwise procedure was applied, with criteria for variable inclusion set at F ≥ 4 and for removal at F < 3. These thresholds ensured an appropriate balance between eliminating weak predictors and avoiding the premature exclusion of variables with potential predictive relevance. The selected criteria correspond approximately to significance levels of *p* = 0.05 and *p* = 0.10 and are consistent with commonly used approaches in regression modeling. This methodology is supported by classical literature on regression analysis (Harrell, 2010; Draper & Smith, 1981).

A maximum of 30 steps was specified for the procedure; however, it was terminated earlier if none of the remaining variables met the criterion for removal. Comparisons between successive models were conducted using the F-test for model reduction, assessing the statistical significance of the regression coefficients.

## 3. Results

A total of 310 respondents participated in the study, aged between 15 and 77 years, with a mean age of 38.06 ± 13.87 years. The age of family members diagnosed with ASD ranged from 1 to 74 years, with the central 50% of values (interquartile range) between 9 and 24 years.

The majority of participants were female (77.74%). In relation to the respondents, individuals with ASD were most commonly their children (27.10%) or siblings (23.87%).

Detailed characteristics of the study participants are presented in Table 1.

The level of depression, anxiety, and stress among respondents was highly variable. The median scores for depression, anxiety, and stress were 16, 12, and 18, respectively, with the central 50% of values falling within the interquartile ranges (Q1–Q3) (Table 2).

In Table 3, the severity of depression, anxiety, and stress is presented. Severe and extremely severe levels were most frequently observed for anxiety (41.29%), followed by depression (35.48%), and least frequently for stress (27.74%). In contrast, normal and mild levels were more common for stress (51.93%) than for depression (42.62%) and anxiety (42.26%).

It was found that respondent age, age of the family member with ASD, education level, and average monthly income per capita significantly influenced the level of depression. The severity of depressive symptoms decreased with increasing respondent age (b = −0.202, *p* < 0.001) and was lower among individuals with the highest income compared to those with the lowest income (b = −3.795, *p* = 0.006). In contrast, depressive symptoms increased with the age of the family member with ASD (b = 0.049, *p* = 0.036) and were higher among individuals with vocational (b = 5.297, *p* = 0.036) and secondary education (b = 2.664, *p* = 0.044) compared to those with primary education. Time since ASD diagnosis was also included in the model but was not a significant predictor of depression.

The model explained 13% of the variance in depression levels (R^2^ = 0.13; adjusted R^2^ = 0.12), indicating limited explanatory power. The RMSE value of 10.49 suggests moderate prediction accuracy.

**Figure 1:**
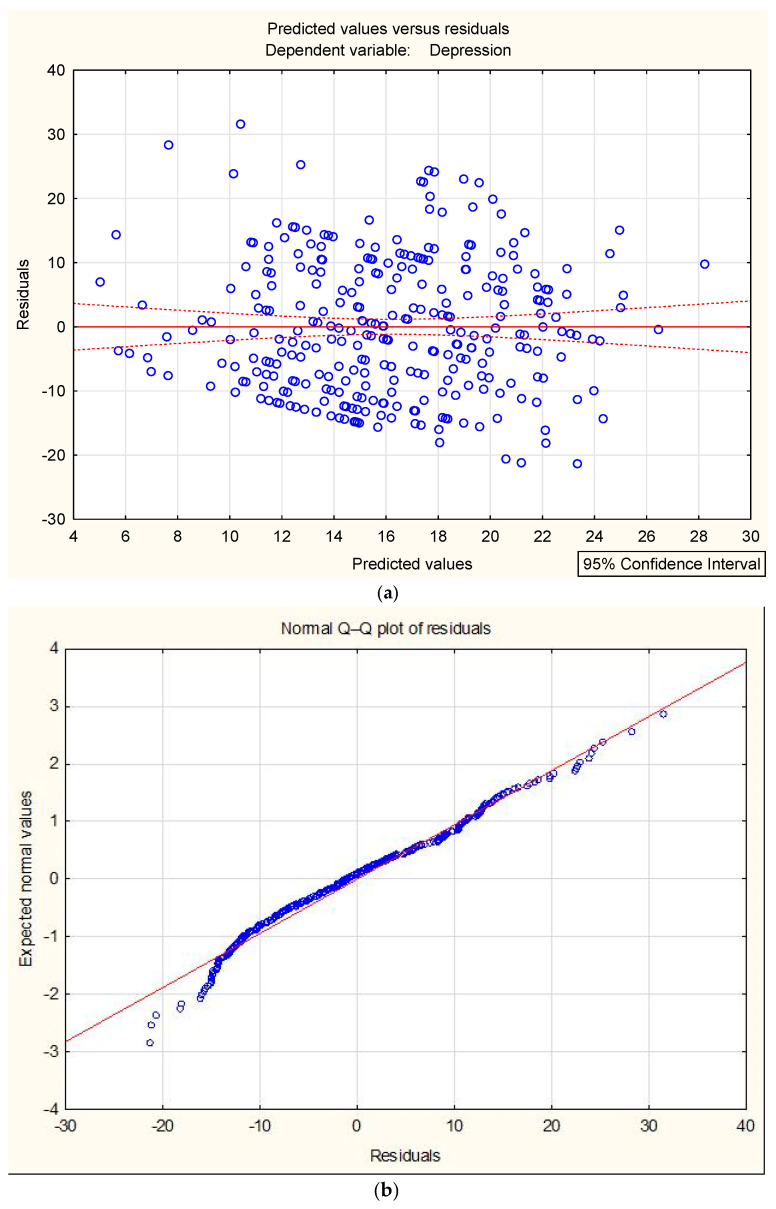
(**a**) Residuals versus predicted values for the linear regression model of depression (DASS-21). (**b**) Normal Q–Q plot of residuals for the depression regression model (DASS-21).

The Durbin–Watson statistic (DW = 1.90) indicated no autocorrelation of residuals. All variance inflation factor (VIF) values ranged between 1.01 and 1.15, suggesting no multicollinearity; therefore, all independent variables were retained in the model. Residual analysis showed no significant deviations from assumptions of linearity or homoscedasticity, with residuals randomly dispersed around zero. No influential outliers were identified. The Q–Q plot indicated slight deviations from normality in the tails, with good fit in the central distribution (Table 4, Figure 1a,b).

Respondent age, degree of kinship, education level, and average monthly income were found to significantly influence anxiety levels. Anxiety symptoms decreased with increasing respondent age (b = −0.202, *p* < 0.001) and were lower among individuals with higher education compared to those with primary education (b = −2.679, *p* = 0.029), as well as among those with incomes of 1501–2500 (b = −3.247; *p* = 0.030) and >4500 (b = −3.573, *p* = 0.008) compared to the lowest income group. Anxiety levels were higher when the individual with ASD was a sibling (b = 3.530, *p* = 0.013) or a parent (b = 5.398, *p* = 0.037), compared to when they were a child. Time since diagnosis and vocational vs. primary education were included in the model but were not statistically significant predictors.

The model explained 18% of the variance in anxiety levels (R^2^ = 0.18; adjusted R^2^ = 0.16), indicating limited explanatory power. The RMSE value of 9.68 suggests moderate predictive accuracy.

**Figure 2:**
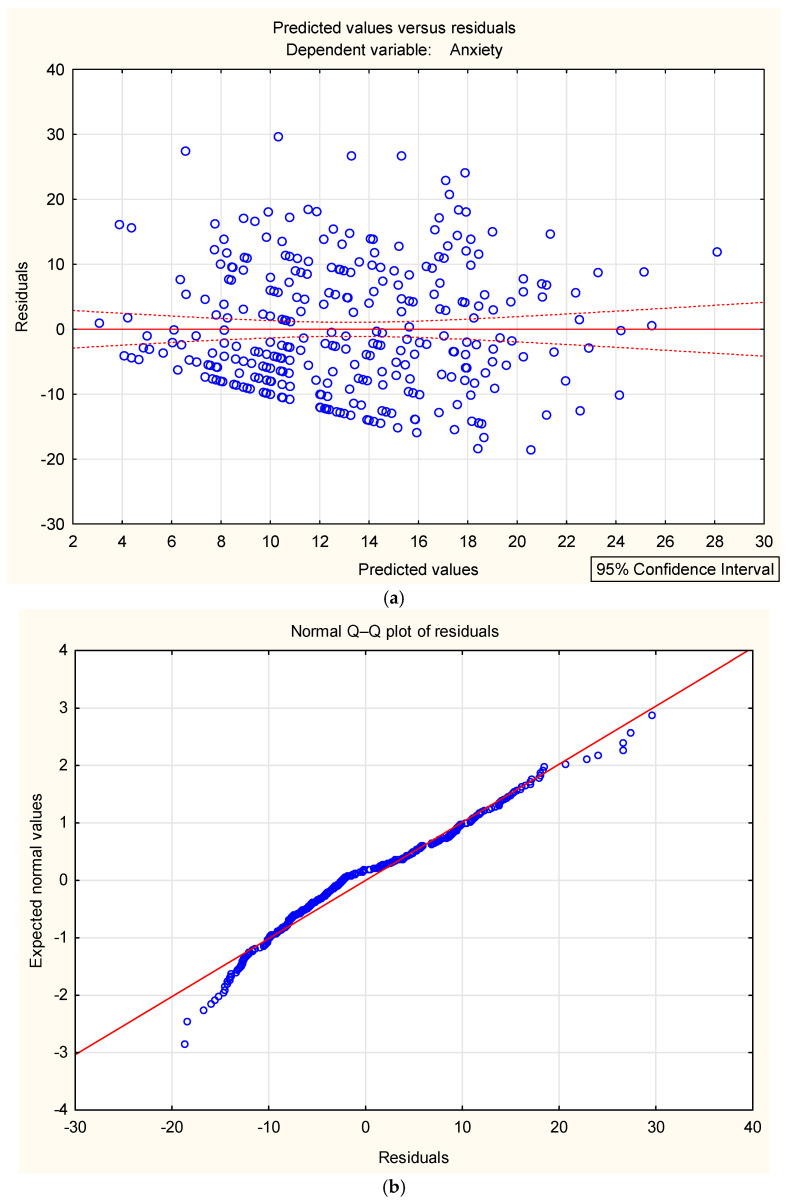
(**a**) Residuals vs. predicted values plot for the anxiety regression model. (**b**) Normal Q–Q plot of residuals for the anxiety regression model.

The Durbin–Watson statistic (DW = 1.77) indicated no autocorrelation of residuals. VIF values ranged from 1.03 to 1.22, indicating no multicollinearity. Residual plots did not show significant violations of linearity assumptions, although slight deviations from homoscedasticity were observed. The Q–Q plot indicated minor deviations from normality at the extremes, with good fit in the central part of the distribution (Table 5, Figure 2a,b).

Respondent age, degree of kinship, and employment status were found to significantly influence stress levels. Stress symptoms decreased with increasing respondent age (b = −0.228, *p* < 0.001) and were lower among employed individuals compared to students (b = −4.781, *p* = 0.026). Stress levels were higher when the individual with ASD was a parent (b = 4.929, *p* = 0.041) compared to when they were a child. Higher education and higher income were included in the model but were not statistically significant predictors.

The model explained 13% of the variance in stress levels (R^2^ = 0.13; adjusted R^2^ = 0.12), indicating limited explanatory power. The RMSE value of 9.68 suggests moderate predictive accuracy.

**Figure 3:**
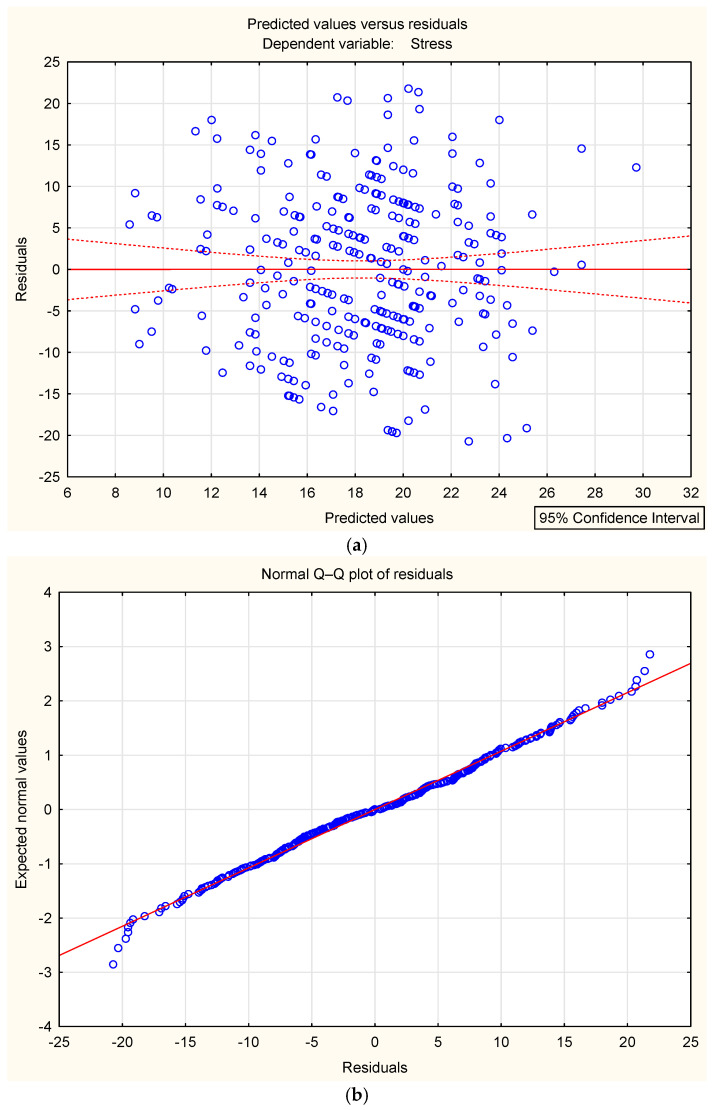
(**a**) Residuals vs. predicted values plot for the stress regression model. (**b**) Normal Q–Q plot of residuals for the stress regression model.

All assumptions of linear regression were met: no autocorrelation of residuals (DW = 1.86), no multicollinearity (VIF: 1.01–1.27), and no significant deviations from linearity, homoscedasticity, or normality based on residual analysis (Table 6, Figure 3a,b).

## 4. Discussion

The results of this study confirm that family members of individuals with autism spectrum disorder (ASD) experience a significant level of psychological distress, including symptoms of depression, anxiety, and stress. Particularly noteworthy is the high prevalence of severe and extremely severe anxiety symptoms observed in the study group. Although the DASS-21 is not a diagnostic instrument, this finding suggests a substantial emotional burden and may indicate an increased need for psychological assessment and support among family members of individuals with ASD. The elevated level of anxiety may reflect chronic uncertainty related to daily functioning, access to support, and concerns about the future of the individual with ASD. These findings are consistent with meta-analyses indicating an increased prevalence of mental health disorders among parents of children with ASD, as well as with empirical studies documenting high levels of depression, anxiety, and stress in this population (Wang, 2021; Li et al., 2022). However, the high prevalence of severe and extremely severe anxiety symptoms observed in the present study should be interpreted with caution. In addition to the psychological burden associated with caring for a family member with ASD, this finding may also reflect characteristics of the study sample, including voluntary participation in an online survey and the possibility of self-selection bias. Therefore, the observed prevalence should not be considered representative of all family members of individuals with ASD. These findings may be interpreted in the context of challenges previously described in the literature, including barriers to accessing healthcare and support services.

Respondent age was a significant factor associated with levels of psychological distress. Younger individuals reported higher levels of depression, anxiety, and stress, which may reflect fewer adaptive resources and greater vulnerability to emotional burden in the early stages of coping with an ASD diagnosis within the family. Similar findings have been reported in previous studies, showing that younger caregivers tend to experience higher stress levels and poorer mental health outcomes (Agarwal et al., 2022; Alibekova et al., 2022). Although these findings should be interpreted with caution, they suggest that younger family members may be at increased risk of psychological distress.

The findings also highlight the importance of socio-economic factors. Lower income was associated with higher levels of depression and anxiety, suggesting that socio-economic inequalities may contribute to psychological distress among family members of individuals with ASD. Previous research has shown that higher income and education levels are associated with better mental health and greater access to social support among caregivers (Li et al., 2022; Watson et al., 2021), while limited financial resources have been linked to higher stress levels and reduced access to supportive services (Watson et al., 2021). Although healthcare system factors were not directly assessed in the present study, these findings may be considered alongside previous evidence indicating that socio-economic inequalities can influence access to available support and therapeutic services.

The analysis revealed significant differences in anxiety and stress levels depending on the degree of kinship with the individual with ASD. Higher levels of distress were observed among individuals for whom the person with ASD was a sibling or a parent, compared to those for whom the individual was a child. This finding suggests that the psychological burden associated with ASD may extend beyond parents and affect the broader family system. Previous studies indicate that siblings of individuals with ASD may experience reduced psychological well-being, increased emotional strain, and limitations in social functioning (Quatrosi et al., 2023). These findings may be relevant for the development of future support strategies; however, they should be interpreted in light of the heterogeneous nature of the study population. The category of “family members” included individuals occupying different family roles (e.g., parents, siblings, grandparents, and other relatives) and living in diverse life circumstances. Consequently, caregiving responsibilities, psychological burden, and support needs may differ substantially across these groups. Future studies should examine these populations separately to better understand role-specific patterns of psychological distress. Accordingly, the present findings should not be generalized to all family members of individuals with ASD.

The relationships observed with respect to education suggest its complex role. Higher education was associated with lower levels of anxiety, which may reflect greater health literacy and better access to information and support. However, in the case of depression, the relationship was less clear. The literature indicates that education may act both as a protective factor and as a factor increasing perceived burden due to greater awareness of the condition (Li et al., 2022; Watson et al., 2021). These findings are consistent with previous evidence suggesting that health literacy may facilitate access to available information and support resources.

Factors related to the individual with ASD also played a significant role. Increasing age of the person with ASD was associated with higher levels of depression among respondents, which may reflect growing concerns about future independence and functioning in adulthood. Research indicates that the transition to adulthood is a particularly challenging period for families of individuals with ASD, associated with sustained high levels of stress and anxiety (Dückert et al., 2023; DesChamps et al., 2020). Previous studies have also described challenges related to the availability and continuity of support for adults with ASD; however, these aspects were not directly examined in the present study.

In this study, time since diagnosis was not a significant predictor of depression, suggesting that adaptation to diagnosis is not a linear process. The literature indicates that stress among parents and caregivers may remain high even many years after diagnosis and depends on multiple contextual factors (DesChamps et al., 2020). Although the present study did not assess long-term service provision or psychological support, these findings underscore the need for further research into factors that may influence the long-term well-being of families of individuals with ASD.

The relatively low coefficients of determination (R^2^ = 0.13–0.18) indicate that a substantial proportion of the variance in psychological distress remains unexplained by the variables included in the present models. This suggests that additional psychosocial factors not assessed in this study, such as social support, coping strategies, caregiving burden, family functioning, or ASD symptom severity, may also play an important role in shaping psychological distress among family members of individuals with ASD. In particular, social support has been identified as a key protective factor reducing symptoms of depression and anxiety (Lei & Kantor, 2021). Accordingly, the identified associations should not be interpreted as a comprehensive explanation of psychological distress among family members of individuals with ASD but rather as evidence of selected socio-demographic and family-related factors associated with psychological distress in this heterogeneous population. Future studies should examine the role of social support, clinical characteristics, and contextual factors, including access to support services, in shaping psychological distress among family members of individuals with ASD.

From a public health perspective, the findings may help identify groups potentially at greater risk of psychological distress, such as younger individuals, those with lower socio-economic status, and family members occupying different roles within the family system. These findings may inform the development of targeted support strategies for families of individuals with ASD. Broader implications for healthcare organization and service provision should be interpreted cautiously, as healthcare-system factors were not directly assessed in the present study. From a broader perspective, coordinating actions across healthcare, education, science and higher education, socio-professional activation, and social welfare systems has long constituted a paramount challenge for policymakers. In essence, this task represents the fundamental creation of a cohesive public policy and should be conceptualized in terms of state obligation. Over a decade ago, the relevant literature highlighted the imperative to initiate comprehensive efforts toward developing an integrated public policy in Poland on this matter, concurrently characterizing the contemporary support system for individuals with autism and their families as highly deficient. Even then, scholars underscored the profound inconsistency and fragmentation of the regulatory legal frameworks (Augustowski et al., 2009). Another prominent example is the Position Statement of the Institute of Healthcare Management on the early diagnosis of autism spectrum disorders in children, which explicitly notes a critical lack of nationwide, institutionalized solutions designed to provide parents and caregivers of children diagnosed with autism spectrum disorders with comprehensive information regarding subsequent clinical and developmental pathways, available support centers, scientifically validated therapeutic methodologies and programs, as well as statutory entitlements within the healthcare, education, and social security systems (Instytut Ochrony Zdrowia, 2025). Consequently, future research should urgently investigate the underlying causes of this systemic inertia. This status quo is undoubtedly exacerbated by institutional siloing within the state apparatus (Sakowicz, 2026). In practice, this phenomenon manifests primarily as a pervasive lack of inter-ministerial coordination, frequent jurisdictional conflicts, and a resulting inefficiency and fragmentation of public policies. Furthermore, the level of stakeholder participation remains pivotal, with the lived experiences of parents (Pisula et al., 2025) and patient advocacy organizations (Porozumienie Autyzm-Polska, 2026) being of critical importance in this context.

Shifting the focus exclusively to the healthcare sector, the most formidable challenges encompass the acute shortage of medical specialists—particularly child and adolescent psychiatrists—regional disparities in access to healthcare services funded by the National Health Fund (NFZ), inadequate tariffication of these services, and the aforementioned fragmented nature of specialized care. These constraints dictate system-level variables, such as actual access to medical services and prolonged waiting times (Narodowy Fundusz Zdrowia, 2026a), ultimately compelling families to utilize private healthcare sectors (Hukałowicz, 2026). Concurrently, it is crucial to emphasize the dynamic escalation in the volume of patients receiving services with a primary or comorbid diagnosis of autism (ICD-10 codes: F84.0, F84.1) or Asperger’s syndrome (ICD-10 code: F84.5) (Narodowy Fundusz Zdrowia, 2026b). As documented in the literature, access to diagnostic procedures in Poland remains strictly limited and unequally distributed across geographical regions and socioeconomic strata. It is widely stressed that public-funded diagnostic assessments under the NFZ are poorly accessible and typically associated with protracted waiting periods.

Several limitations should be considered when interpreting the findings. First, all data, including the ASD diagnosis, were based on respondents’ self-reports and were not independently verified. Second, due to the anonymous nature of the survey, it was not possible to determine whether more than one participant came from the same family, which may have affected the independence of observations. Third, the use of an online CAWI survey and voluntary participation may have introduced selection bias, as individuals with limited internet access, lower digital literacy, or lower socioeconomic status may have been underrepresented. Furthermore, detailed information regarding the recruitment flow was not available from the panel provider; therefore, the representativeness of the final study sample cannot be fully assessed. Fourth, several potentially important variables, including ASD symptom severity, co-occurring intellectual disability, caregiving burden, and social support, were not assessed and may have influenced the observed associations. Finally, the study population was heterogeneous with regard to both the type of family relationship and the age of the individual with ASD. Parents, siblings, grandparents, and other relatives may differ substantially in caregiving responsibilities, psychological burden, coping strategies, and support needs, while families of young children and adults with ASD may face different challenges. Accordingly, the findings should be interpreted as exploratory and should not be generalized equally across all groups of family members of individuals with ASD. Future studies should examine more homogeneous populations and incorporate clinical, caregiving-related, and healthcare-related characteristics to better explain the factors associated with psychological distress (Otrębski et al., 2022).

## 5. Conclusions

Psychological distress appears to be an important issue among various groups of family members of individuals with ASD, although the nature and extent of this burden may differ depending on family role and life circumstances. Higher levels of depression, anxiety, and stress observed among younger individuals and those with lower socio-economic status highlight the importance of socio-economic inequalities.

The findings support the importance of a family-centered approach to supporting individuals with ASD and their relatives. Broader healthcare-system implications should be interpreted cautiously, as healthcare system factors were not directly examined in this study. Psychological burden affects not only parents but also other family members, who are often not included in formal support structures.

The results may help identify characteristics associated with increased psychological distress within a heterogeneous population of family members of individuals with ASD and may inform future research and the development of targeted support strategies. Further studies incorporating healthcare system factors, caregiving burden, social support, and clinical characteristics are needed to better understand the determinants of psychological distress in this population.

## Figures and Tables

**Table 1:** Characteristics of respondents related to individuals with ASD.

Variable	Category		n	%
Gender	Female		241	77.74
	Male		65	20.97
	Other		1	0.32
	Prefer not to say		3	0.97
Relationship to person with ASD	Child		84	27.10
	Sibling		74	23.87
	Parent		16	5.16
	Grandchild		23	7.42
	Other relatives		113	36.45
Education level	Primary		11	3.55
	Vocational		21	6.77
	Secondary		123	39.68
	Higher		155	50.00
Employment status	Student		26	8.39
	Employed		213	68.71
	Retired/Pensioner		40	12.90
	Unemployed		31	10.00
Monthly income per capita	<1500 PLN		32	10.32
	1501–2500 PLN		61	19.68
	2501–3500 PLN		57	18.39
	3501–4500 PLN		65	20.97
	>4500 PLN		95	30.65
Time since ASD diagnosis	<1 year		53	17.10
	1–5 years		162	52.26
	6–10 years		51	16.45
	>10 years		44	14.19
Continuous variables:				
Variable	Mean (M)	SD	Median (Me)	Range
Age of respondent	38.06	13.87	35.0 (28–46)	15–77
Age of family member with ASD	17.91	12.75	14.0 (9–24)	1–74

**Table 2:** Levels of depression, anxiety, and stress (DASS-21).

Scale	Mean	SD	Min	Max	Median	Q1	Q3
Depression	16.18	11.25	0.0	42.0	16.0	6.0	26.0
Anxiety	13.19	10.70	0.0	42.0	12.0	4.0	22.0
Stress	18.24	9.90	0.0	42.0	18.0	12.0	26.0

**Table 3:** Severity of depression, anxiety, and stress (DASS-21).

Severity	Depression n (%)	Anxiety n (%)	Stress n (%)
Normal	96 (30.97)	119 (38.39)	125 (40.32)
Mild	33 (10.65)	12 (3.87)	36 (11.61)
Moderate	71 (22.90)	51 (16.45)	63 (20.32)
Severe	48 (15.48)	31 (10.00)	70 (22.58)
Extremely severe	62 (20.00)	97 (31.29)	16 (5.16)

**Table 4:** Factors associated with depression among family members of individuals with ASD (N = 310).

Variable	β (Standardized)	SE (β)	b	SE (b)	t	*p*-Value
Intercept	—	—	22.274	2.038	10.929	<0.001
Age of respondent	−0.249	0.054	−0.202	0.044	−4.642	<0.001
Age of family member with ASD	0.116	0.055	0.103	0.049	2.103	0.036
Time since diagnosis (>10 years vs. <1 year)	−0.105	0.056	−3.373	1.799	−1.875	0.062
Vocational vs. primary education	0.118	0.056	5.297	2.513	2.108	0.036
Secondary vs. primary education	0.116	0.057	2.664	1.316	2.025	0.044
Income >4500 vs. <1500	−0.156	0.056	−3.795	1.365	−2.780	0.006
Model summary:						
	R = 0.369	R^2^ = 0.136	Adjusted R^2^ = 0.119			
	F(6, 303) = 7.94	*p* < 0.00001				
	RMSE = 10.44					

**Table 5:** Factors associated with anxiety among family members of individuals with ASD (N = 310).

Variable	β (Standardized)	SE (β)	b	SE (b)	t	*p*-Value
Intercept	—	—	21.240	2.062	10.299	<0.001
Age of respondent	−0.202	0.056	−0.156	0.043	−3.594	<0.001
Time since diagnosis (>10 vs. <1 year)	−0.104	0.053	−3.183	1.625	−1.958	0.051
Sibling vs. child	0.141	0.057	3.530	1.419	2.487	0.013
Parent vs. child	0.112	0.053	5.398	2.579	2.093	0.037
Vocational vs. primary education	0.101	0.055	4.274	2.334	1.831	0.068
Higher vs. primary education	−0.125	0.057	−2.679	1.223	−2.191	0.029
Income 1501–2500 vs. <1500	−0.121	0.056	−3.247	1.492	−2.177	0.030
Income >4500 vs. <1500	−0.154	0.058	−3.573	1.337	−2.673	0.008
Model summary:						
	R = 0.423	R^2^ = 0.179	Adjusted R^2^ = 0.157			
	F(8, 301) = 8.21	*p* < 0.00001				
	RMSE = 9.68					

**Table 6:** Factors associated with stress among family members of individuals with ASD (N = 310).

Variable	β (Standardized)	SE (β)	b	SE (b)	t	*p*-Value
Intercept	—	—	28.899	1.887	15.314	<0.001
Age of respondent	−0.320	0.058	−0.228	0.041	−5.497	<0.001
Parent vs. child	0.110	0.054	4.929	2.396	2.057	0.041
Higher vs. primary education	−0.113	0.058	−2.233	1.148	−1.945	0.053
Employed vs. student	−0.134	0.060	−4.781	2.140	−2.234	0.026
Income >4500 vs. <1500	−0.109	0.056	−2.327	1.206	−1.930	0.055
Model summary:						
	R = 0.365	R^2^ = 0.133	Adjusted R^2^ = 0.119			
	F(5, 304) = 9.32	*p* < 0.00001				
	RMSE = 9.68					

## Data Availability

The original contributions presented in this study are included in the article. Further inquiries can be directed to the corresponding author.
